# Lectures on Renal and Urinary Diseases

**Published:** 1897-11

**Authors:** 


LECTURES ON RENAL AND URINARY DISEASES. By Robert Saundby, M. D., Edin., Fellow of the Royal College of Physicians, London; Emeritus Senior President of the Royal Medical Society; Fellow of the Royal Medico-Chirurgical Society; Professor of Medicine in Mason College, Birmingham, etc., etc., etc. Second edition, 434 pages, and numerous illustrations. Price, in cloth, $2.50 net. W. B. Saunders, publishers, 925 Walnut St., Philadelphia, 1897.

This book is really a revised one volume edition of the author's two books, the one on Bright's Disease, and the other on Diabetes, to which has been added some chapters on "Miscellaneous Renal Diseases." The first section of this volume, embracing 178 pages, is devoted to the discussion of Bright's Disease, under the following sub-headings: General anatomy of the kidney, the pathology of albuminuria, the pathology of dropsy, pathological relations of tube casts, cardio vascular changes, pathology of polyuria, pathology of uræmria; retinal changes, history, classification, etiology; infective nephritis, lithæmic nephritis, obstructive nephritis, complications of Bright's disease, treatment of Bright's disease. The second section, covering 38 pages, treats of the clinical examination of the urine. Section third, is made up of a discussion of diabetes, including diabetes mellitus, etiology, morbid anatomy and clinical history; diabetic coma, treatment of diabetes, and diabetes insipidus. The fourth section discusses stone in the kidney, hydronephrosis, pyonephrosis, pyelitis, hæmaturia, and hæmoglobinuria.

The many years of special study which Dr. Saundby has given to these diseases thoroughly qualifies him for the task of writing an authoritative work. The combination of the two former volumes into one is quite an advantage and will increase the popularity of the book. This volume is one of the best extant on the subjects of which it treats. S. E. H.

